# Statin Intolerance—We Know Everything, We Know Nothing

**DOI:** 10.3390/jcm11175250

**Published:** 2022-09-05

**Authors:** Maciej Banach

**Affiliations:** 1 Department of Preventive Cardiology and Lipidology, Medical University of Lodz (MUL), 93338 Lodz, Poland; maciej.banach@icloud.com; 2 Department of Cardiology and Congenital Diseases of Adults, Polish Mother’s Memorial Hospital Research Institute (PMMHRI), 93338 Lodz, Poland; 3 Cardiovascular Research Centre, University of Zielona Gora, 65046 Zielona Gora, Poland

Lipid disorders are the most common risk factors of cardiovascular disease (CVD) [1] and, more importantly, the worst monitored and controlled, with only a third of patients being on the low-density lipoprotein cholesterol (LDL-C) target in Europe [2], and only 24% in the Central and Eastern European (CEE) countries [3]. The coronavirus disease 2019 (COVID-19) pandemic has made this situation even worse; therefore, an urgent call for action is required to be effective in primary and secondary preventions to delay or avoid the first event or to prevent recurrent CVD events [4,5,6].

One of the reasons of this ineffectiveness is therapy non-adherence, and the most important reason for non-adherence is statin-associated side effects, mainly including statin-associated muscle symptoms (SAMS) that is generally called statin intolerance (SI) [7]. In fact, in 2022, considering our knowledge, diagnostics methods and availability of drugs [7], we should not talk about statin intolerance anymore, and it should not also be a subject of the anniversary Special Issue of the *Journal of Clinical Medicine*. Unfortunately, the situation is completely different, and statins are still mostly used in mainly low to moderate doses; even in patients at very high and extremely high cardiovascular risk [6,8], high-intensive statin therapy is only used in about 5% [6], and statin intolerance in everyday clinical practice and in observational cohort studies and registries is still highly overdiagnosed with a prevalence of even 30% and more [9]. In most of SI patients, statins are immediately discontinued without any attempt to assess and exclude risk factors (e.g., drugs, alcoholism, intensive exercise and low vitamin D) or conditions (hypothyroidism, obesity, diabetes, chronic kidney disease and chronic liver disease) that might significantly increase the SI risk [9,10,11]. A simple question on regular exercise [12], medications taken and/or an optimization of the therapy of coexistent diseases can indeed significantly decrease SAMS’s prevalence [13]. We should also always remember about the nocebo effect or more correctly called the drucebo effect (as it is directly linked to statin therapy)—the psychological attitude that adverse effects after statins will certainly appear [14,15,16]. Based on our data, 50–70% of SI cases [14] and even 90% and more based on the n-of-1 trials [16,17] might be due to the nocebo/drucebo effect. It is worth emphasizing that we already know how to diagnose the nocebo/drucebo effect; the exclusion of SI risk factors, the application of the SAMS Clinical Index score and continuous patient education might be of large help with this [13].

In all those with confirmed statin intolerance (it is only 9.1% or less <7% if diagnosed with the approved definitions [9] and only about 2% for the confirmed complete statin intolerance diagnosis [18]), we are not allowed to discontinue statin therapy (unless creatine kinase (despite it is not a very sensitive biomarker for SI) is >4 ULN and might be associated with the risk of myopathy/myonecrosis or the pain is intolerable for the patients [13]), and we should try to convince the patient to continue statin therapy at lower doses (accompanied by adding ezetimibe, depending on the risk—preferably in the form of polypill); change the statin preparation (preferably to hydrophilic statins or pitavastatin, which based on still limited data has the same risk of SI like placebo); to consider alternate-day statin therapy (even 2–3 times a week; we have data for rosuvastatin and atorvastatin suggesting that such management is still effective in LDL-C reduction); or to consider non-statin drugs, including bempedoic acid, PCSK9 inhibitors, inclisiran or even fibrates and nutraceuticals [6,13,19,20,21,22,23]. The most important aim for patients who often have high baseline levels of LDL-C (even 140–160 mg/dL (3.6–4.1 mmol/L) and higher) and are at high and very high CVD risk is to have them on LDL-C targets, as those patients, based on available data, are at a very high risk of cardiovascular events [18,24].

Based on the abovementioned information, statin intolerance is still a great challenge in the clinical setting, and there are still many unsolved issues concerning the mechanisms, definition, diagnosis and management. Therefore, in this anniversary Special Issue, we would like to invite you to submit your papers on new biomarkers that may help in diagnosing and/or differentiating SI in practical algorithms/scores and/or IT software (with the application of innovative tools such as AI and machine/deep learning) that may help exclude the nocebo/drucebo effect and confirm the diagnosis of statin intolerance and real-world data on the prevalence of SI, its diagnosis and effectiveness of available therapies in different populations. RWD in the context of statin intolerance seems to be critical, because RCTs cannot present the real prevalence of statin intolerance (due to study’s design and exclusion of many risk factors/conditions), despite the fact that they may show the risk and the pathomechanism of de novo SAMS in highly selected patients who are optimally monitored and treated, and this is obviously also of great value [25,26]. I am looking forward to high-quality papers, and after acceptance, I would like to discuss the publication of the papers on the journal’s webpage during short webinars.
